# Clinical and Metabolic Characterization of Women With Gestational Diabetes Mellitus Within the First Year Postpartum

**DOI:** 10.1210/jendso/bvae044

**Published:** 2024-03-12

**Authors:** Laura Løftgaard Knudsen, Sine Knorr, Susanne Kastberg Prange, Charlotte Wolff, Helle Nørgaard, Anne Mette Torp, Lene Ring Madsen, Lene Mortensen, Henrik Holm Thomsen, Lars Peter Sørensen, Per Glud Ovesen, Jens Fuglsang, Ulla Kampmann

**Affiliations:** Steno Diabetes Center Aarhus, Aarhus University Hospital, Aarhus, Denmark; Steno Diabetes Center Aarhus, Aarhus University Hospital, Aarhus, Denmark; Department of Clinical Medicine, Aarhus University, Aarhus, Denmark; Steno Diabetes Center Aarhus, Aarhus University Hospital, Aarhus, Denmark; Department of Gynecology and Obstetrics, Aarhus University Hospital, Aarhus, Denmark; Steno Diabetes Center Aarhus, Aarhus University Hospital, Aarhus, Denmark; Steno Diabetes Center Aarhus, Aarhus University Hospital, Aarhus, Denmark; Steno Diabetes Center Aarhus, Aarhus University Hospital, Aarhus, Denmark; Department of Internal Medicine, Gødstrup Hospital, Herning, Denmark; Danish Diabetes Academy, Odense University Hospital, Odense, Denmark; Department of Internal Medicine, Horsens Regional Hospital, Horsens, Denmark; Department of Clinical Medicine, Aarhus University, Aarhus, Denmark; Department of Internal Medicine, Viborg Regional Hospital, Viborg, Denmark; Department of Internal Medicine, Randers Regional Hospital, Randers, Denmark; Steno Diabetes Center Aarhus, Aarhus University Hospital, Aarhus, Denmark; Department of Clinical Medicine, Aarhus University, Aarhus, Denmark; Department of Gynecology and Obstetrics, Aarhus University Hospital, Aarhus, Denmark; Department of Clinical Medicine, Aarhus University, Aarhus, Denmark; Department of Gynecology and Obstetrics, Aarhus University Hospital, Aarhus, Denmark; Steno Diabetes Center Aarhus, Aarhus University Hospital, Aarhus, Denmark; Department of Clinical Medicine, Aarhus University, Aarhus, Denmark

**Keywords:** diabetes, gestational diabetes mellitus, oral glucose tolerance test, prediabetes, pregnancy

## Abstract

**Context:**

Women with gestational diabetes mellitus (GDM) have an increased risk of long-term complications, including impaired glucose metabolism, type 2 diabetes (T2DM), cardiovascular disease, and obesity. In current clinical practice, a 1 size fits all approach to GDM is applied, although heterogeneity among women with GDM has been recognized.

**Objective:**

To give the most adequate preventive care and postpartum (PP) guidance, we aimed to make a metabolic characterization and identify subgroups of women with previous GDM within the first year PP.

**Methods:**

In this prospective cohort study, we collected data in gestational week 34-38, at 3 months, and 1 year PP on women with GDM who participated in a PP follow-up program in Central Region Denmark from April 2019 to December 2022.

**Results:**

In total, 1270 women were included in the program in late pregnancy. Of the 768 women participating in either the oral glucose tolerance test 3 months PP (n = 545) or the 1-year follow-up (n = 493) or both (n = 261), 608 (79.2%) were normoglycemic, 137 (17.8%) had prediabetes, 20 (2.6%) had T2DM, and 3 (.4%) had developed T1DM. More than 40% of the women gained weight in the first year PP compared with their pregestational weight.

**Conclusion:**

Our study shows that 20.8% of women with GDM who volunteered to participate in a clinical follow-up program developed prediabetes or diabetes (T1DM and T2DM) within the first year PP. The GDM diagnosis encompasses a heterogenetic group of women and a deeper characterization may provide an opportunity for a more personalized risk assessment to prevent the progression to T2DM.

Gestational diabetes mellitus (GDM) is defined as any glucose intolerance with onset during pregnancy and with remission upon delivery [1]. Due to the rising prevalence of obesity among women of fertile age, the incidence of GDM rises concomitantly and is the most common medical complication of pregnancy [2]. GDM entails both short-term obstetrical complications related to hyperglycemia and fetal overgrowth, and a substantial long-term risk of maternal diabetes [3] and cardiovascular disease [4]. Recent studies have addressed that GDM is a heterogeneous condition and GDM subtypes have been defined [5-8] according to impaired insulin sensitivity or deficient insulin secretion. Furthermore, Layton et al also found that the lipid profile differs between women with GDM and women with normoglycemia (NG), and that there is a heterogeneity in lipid profiles among the GDM subtypes [9]. In addition, the rise in insulin resistance and the increased demand for insulin during pregnancy, can also unveil an underlying type 1 diabetes (T1DM). Thus, the-one-size-fits-all approach to GDM and postpartum (PP) follow-up in current clinical practice might not be appropriate. Therefore, a thorough subtyping of women with GDM may provide the opportunity for a more personalized risk assessment and treatment both during and after pregnancy. Accordingly, in a large Danish cohort, we aimed to evaluate glycemic status within the first year PP in pregnancies complicated by GDM. Furthermore, we aimed to characterize the women with GDM metabolically and clinically describing glycemic parameters, lipid profiles, inflammatory markers, and GAD65 autoantibody (GAD65Ab) status as well as weight status, both late in third trimester and within the first year PP.

## Materials and Methods

This study is a prospective cohort study based on data from a clinical follow-up program for women with GDM running from April 2019 to December 2022 in the Central Denmark Region. The program aimed to support women with GDM to prevent type 2 diabetes (T2DM). In addition to the standard care for women with GDM, women who entered the program were offered an additional individual consultation at the outpatient clinic at their local department of obstetrics and gynecology in gestational week (GW) 34-38. The consultation, given by a nurse or a dietician, included individual counseling on healthy lifestyle, including advice on diet, physical activity, breastfeeding, and sleep focusing on life following a pregnancy with GDM. In addition, at the GW 34-38 visit, blood and urine samples were collected, blood pressure (BP), height (using a standard stadiometer), and weight were measured. Pregestational BMI (body mass index, kg/m^2^) was calculated from self-reported pregestational weight. Gestational weight gain (GWG) was calculated as the difference between self-reported pregestational weight and weight measured at the GW 34-38 visit.

**Figure 1.**
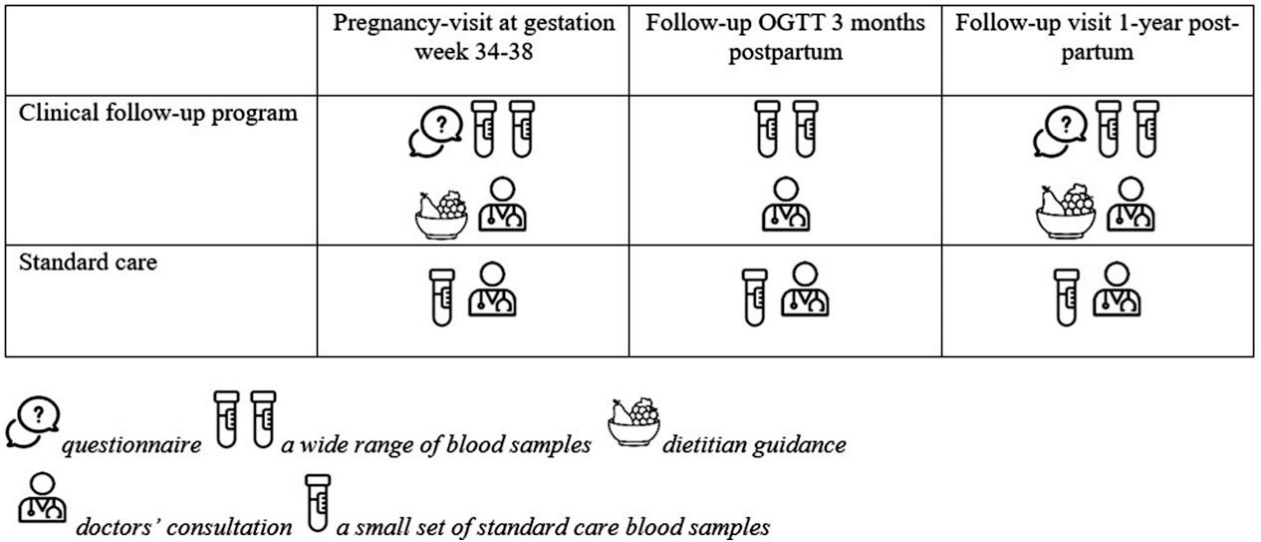
Overview describing standard care and the clinical follow-up program.

**Figure 2.**
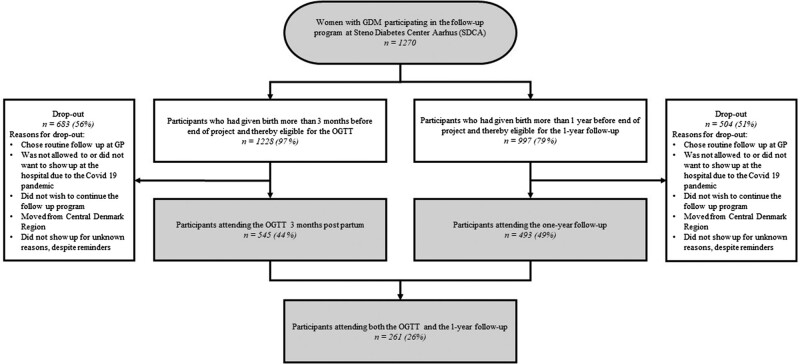
Flow chart of the study population.

Postpartum, women could choose to attend a follow-up program consisting of a 2-hour oral glucose tolerance test (OGTT) and concomitant blood sampling 3 months PP, and/or an individual lifestyle consultation 1 year PP (Fig. 1). At the follow-up visit, 1 year PP blood samples were collected, and BP, height, and weight were measured. Change in BMI was defined as the difference between pregestational BMI and BMI at 1 year PP. In addition, the women were asked to fill out a questionnaire late in pregnancy (GW 34-38) and 1 year PP. The questionnaires consisted of questions regarding family history of diabetes, country of birth, education level, smoking habits, physical activity level, and pregestational BMI. The women invited in the cohort could choose either the individual consultation late in pregnancy, the 3-month follow-up visit, the 1-year follow-up visit, or all 3 visits (Fig. 1). If the woman was not interested in attending the follow-up program, she could freely choose to go to her general practitioner for a checkup, including a measurement of hemoglobin HbA1c and/or an OGTT 3 months PP and every 1 to 3 years thereafter. There were no inclusion or exclusion criteria as the clinical follow-up programs were offered to all women with GDM in the Central Denmark Region, but a woman was eligible to be included in the current study if data on blood samples and questionnaires were available. As the follow-up program was limited to the prespecified period from April 2019 to December 2022, some women were not eligible to participate in the follow-up consultations PP, explaining why participation varied (Fig. 2). The women gave oral consent to participate, but as the project was categorized as a quality assurance project ethics approval was not required. This is according to the GDPR regulation and the Danish data protection legislation.

### Data Collection

All data, including blood test results and questionnaires, were extracted from the electronic patient file system. Data were collected at 5 different hospitals in the Central Denmark Region. Only women with biochemical evaluation at the GW 34-38 visit were included in the baseline analyses. Women with data at either OGTT 3 months PP or 1 year PP were included in the 1-year evaluation.

At baseline, medical history including family history on diabetes (grandparents, parents, siblings of parents, siblings, or children) were reported. Educational level was grouped into elementary school, short-term education, intermediate higher education, long higher education, and other education. Physical activity was defined as moderate to heavy physical activity, for instance, exercise or competitive sports, heavy gardening work, brisk walking, cycling at moderate to fast pace, physical strenuous work for at least 30 minutes per day, with 3 intervals of 10 minutes each acceptable.

Blood samples including proinsulin C-peptide, GAD65Ab, hemoglobin A1c (HbA1c), lipids (total cholesterol, triglyceride, high-density lipoprotein [HDL], and low-density lipoprotein [LDL]), C-reactive protein (CRP), and alanine transaminase (ALAT) were collected both at baseline and at follow-up. Blood samples were collected as nonfasting during pregnancy and as fasting at the OGTT 3 months PP and at the visit 1 year PP.

At 1 year PP, glucose parameters and GAD65Ab were used to divide women into 4 groups: NG, prediabetes mellitus (pDM), T2DM, or T1DM. The criteria for prediabetes were either impaired fasting glucose where fasting plasma glucose (FPG) ≥6.1 to <7 mmol/L (110-126 mg/dL) without impaired glucose tolerance or as impaired glucose tolerance where FPG <7.0 mmol/L (126 mg/dL) and 2-hour glucose ≥7.8 to <11.1 mmol/L (140-199 mg/dL), or HbA1c between 42 and 48 mmol/mol (5.7-6.4%) [1, 10, 11]. The diagnostic threshold for T2DM was based on either FPG ≥7.0 mmol/L (126 mg/dL), OGTT 120 minutes ≥11.1 mmol/L (200 mg/dL), or HbA1c ≥ 48 mmol/mol (≥ 6.5%), and anti-GAD65Ab <10 × 10^3^ international units/L. T1DM was diagnosed using the same thresholds but with the presence of high titers of anti-GAD65Ab > 10 × 10^3^ international units/L [12].

After dividing the women with previous GDM into the 4 groups according to their glycemic status, a metabolic characterization was performed using the available data on blood samples, BP, BMI, height and weight measurements including reach of pregestational weight (±1.0 kg), and GWG recommendations by the Institute of Medicine (IOM) [13]. Information from the questionnaires at the time of diagnosis of either prediabetes or diabetes were also noted.

To assess insulin resistance (IR) and β-cell function (B) homeostatic model assessment for (HOMA)-IR and HOMA-B [14, 15] were calculated based on C-peptide and plasma glucose concentrations using the following equations:


$$\begin{array}{rl} \mathrm{HOMA}\text{-}\mathrm{IR}(\mathrm{CP}) & =1.5+\mathrm{fasting}\;\mathrm{blood}\;\mathrm{glucose}(\mathrm{FBG})\\ & \;\times \mathrm{fasting}\;C\text{-}\mathrm{peptide}(\mathrm{FCP})/2800\\ & \;\mathrm{HOMA}\text{-}B=0.27\times \mathrm{FCP}/(\mathrm{fasting}\;\mathrm{plasma}\;\mathrm{glucose}(\mathrm{FPG})-3.5) \end{array}$$

### Statistics

Categorical variables are reported as n (%) and continuous variables as mean (SD). One-way analysis of variance (ANOVA) was used to test the difference between the groups when the dependent variable was continuous, and if a significant difference was found, a Student's t-test was performed to examine between which groups the difference existed. Data were normally distributed (as assessed by QQ plots (quantile-quantile plot)), had equal SDs, and observations were independent. For categorical variables, a chi^2^ test was performed.

The comparison between clinical characteristics either during the OGTT or at the 1 year PP visit was based on blood sample measurements from the day women were diagnosed with dysglycemia.

For the women who were in the NG group both at OGTT and 1 year PP, the measurements were recorded at the 1 year PP. For the women only participating in either the OGTT or 1 year PP, the measurements would be from that specific visit, where data were available.

To examine whether the study population represented the women not participating, we compared baseline characteristics of women participating in the OGTT and/or the 1 year PP with those who did not.

All data processing and analyses were made using Stata 17 (Stata Corp, College Station, TX, USA). *P* < .05 was considered to be statistically significant.

## Results

### Participation at Baseline and in the Follow-up Program

As showed in Fig. 2, 1270 women with GDM participated in the GW 34-38 visit. Of these women, 1228 were eligible to participate in the follow-up, including OGTT, 3 months PP. Moreover, 997 women were eligible to participate in the 1 year PP follow-up as they had given birth more than 1 year prior to the end of the program.

In total, 545 (44%) women participated in the 3-month PP visit, including the OGTT, and 493 (49%) women participated in the 1 year PP visit, whereas 261 (26%) women participated in both visits. This left 777 individuals for data analyses. Nine (1.2%) women with incomplete data were excluded from the analysis, leaving 768 women as the final study population.

### Baseline Visit

Baseline characteristics of the 1270 women participating in the GW 34-38 visit are shown in Table 1. Overall, women were 33.7 years (4.9) and had a mean BMI prior to pregnancy of 28.2 kg/m^2^ (6.4). The only antidiabetic treatment used was insulin, and 12% of the women were treated with insulin during pregnancy with a daily dose late in pregnancy of 36.2 (25.3) international units (IU), corresponding to .37 (.24) IU/kg. The majority (68.3%) of women had an intermediate level education (eg, nurse or teacher) or less. About half were primipara and 3 out of every 4 were of European descent. Approximately 37% had a family history of diabetes (T1DM, T2DM, or other not specified).

**Table 1.** Baseline clinical characteristics of the participants (n = 1270) at the first project visit (pregnancy visit)

Age (years)	33.7 (4.9)
Gestation week at pregnancy visit (week)	36.3 (1.9)
Blood pressure	
Systolic (mmHg)	121.5 (12.0)
Diastolic (mmHg)	79.3 (22.2)
Pregestational weight (kg)	77.8 (19.0)
Pregestational BMI (kg/m^2^)	28.2 (6.4)
Underweight (BMI <18.5), n (%)	20 (1.6)
Normal weight (BMI ≥18.5-<25), n (%)	283 (22.3)
Overweight (BMI ≥25-<30), n (%)	278 (21.9)
Obese (BMI ≥30), n (%)	287 (22.6)
Missing, n (%)	402 (31.6)
Gestational weight gain (kg)	10.0 (7.7)
Underweight (pregestational BMI <18.5) (kg)	14.8 (7.1)
Normal weight (pregestational BMI ≥18.5-<25) (kg)	12.6 (5.8)
Overweight (pregestational BMI ≥25-<30) (kg)	10.5 (6.7)
Obese (pregestational BMI ≥30) (kg)	7.2 (9.6)
Followed the recommendations regarding GWG set by IOM	
Yes, n (%)	270 (21.3)
No, gained more weight, n (%)	269 (21.2)
No, gained less weight, n (%)	280 (22.0)
Missing, n (%)	451 (35.5)
Insulin treated during pregnancy, n (%)	153 (12.0)
Insulin dose (daily dose, IU)	36.2 (25.3)
Insulin dose (IU/kg)	.37 (.24)
**Biochemical parameters**	
C-peptide (370-1470 pmol/L)	2159.3 (1201.5)
Anti-Gad65ab (10^3^ int. U/L)	
Anti-Gad65ab (10^3^ int. U/L) <10, n(%)	1075 (84.6)
Anti-Gad65ab (10^3^ int. U/L) >10, n(%)	35 (2.8)
Missing, n (%)	160 (12.6)
HOMA-B	348.4 (220.6)
HOMA-IR	6.0 (3.6)
Plasma-glucose (4.2-7.8 mmol/L)	5.5 (1.2)
HbA1c (<48 mmol/mol)	35.4 (4.5)
B-hemoglobin (7.0-9.1 mmol/L)*^a^*	7.7 (.9)
Triglyceride (1.33-4.72 mmol/mol)*^a^*	3.4 (1.2)
Cholesterol	
Total cholesterol (4.4-8.8 mmol/L)*^a^*	6.1 (1.2)
HDL (1.2-2.9 mmol/L)*^a^*	1.7 (.4)
LDL (1.6-5.6 mmol/L)*^a^*	3.0 (1.0)
CRP (<8.0 mg/L)	14.2 (25.4)
ALAT (5-42 U/I)*^a^*	28.3 (37.5)
**Background info**	
Education	
Elementary school, n (%)	62 (4.9)
Short-term education, n (%)	379 (29.8)
Intermediate higher education, n (%)	427 (33.6)
Long higher education, n (%)	269 (21.2)
Other (including foreign education), n (%)	42 (3.3)
Missing, n (%)	91 (7.2)
Ethnicity	
Europe, n (%)	963 (75.8)
Asia, n (%)	75 (5.9)
Middle east, n (%)	103 (8.1)
Other, n (%)	34 (2.7)
Missing, n (%)	95 (7.5)
Parity	
Primipara, n (%)	589 (46.4)
Multipara, n (%)	594 (46.8)
Missing, n (%)	87 (6.8)
Family history of diabetes mellitus*^b^*	
Yes, n (%)	468 (36.9)
No, n (%)	499 (39.3)
Missing, n (%)	303 (23.9)
Physical activity*^c^*	
Never physical active, n (%)	65 (5.1)
Physical active 1-3 days/week, n (%)	193 (15.2)
Physical active 4-7 days/week, n (%)	226 (17.8)
Missing, n (%)	786 (61.9)
Smoking	
Never smoked, n (%)	363 (28.6)
Smoked before pregnancy, n (%)	78 (6.1)
Smoking, n (%)	38 (3.0)
Missing, n (%)	791 (62.3)

Data are mean (SD) unless otherwise indicated.

Abbreviations: ALAT, alanine transaminase; B, β-cell function; BMI, body mass index; CRP, C-reactive protein; GWG, gestational weight gain; HDL, high-density lipoprotein; HOMA, homeostatic model assessment; IOM; Institute of Medicine; IR, insulin resistance.

^*a*^Reference interval for pregnant women GW 35-42 [16].

^*b*^Family history of diabetes defined as 1 or more relatives with DM (for further details, see text).

^*c*^Physical activity defined as at least 30 minutes per day (for further details, see text).

### Baseline Data Divided According to Glycemic Status at Follow-up (OGTT 3 Months Postpartum or 1-Year Follow-up Visit)

For the PP data analyses, data from the 3-month visit and 1-year visits were pooled. Of the 768 women participating in either OGTT (n = 545) or 1 year follow-up PP (n = 493) or both (n = 261), 608 (79.2%) were NG, 137 (17.8%) had pDM, 20 (2.6%) had T2DM, and 3 (0.4%) had T1DM (Table 2). Due to the small number, the women diagnosed with T1DM were excluded from further analyses.

**Table 2.** Baseline clinical characteristics depending on glycemic status within the first year

	Normoglycemic (n = 608)	pDM (n = 137)	T2DM (n = 20)	Between groups
Age (years)	33.9 (4.9)	34.7 (4.9)	34.1 (5.0)	NS
Gestation week at pregnancy visit (week)	36.4 (1.5)	35.8 (3.0)	36.4 (1.4)	<.001*^b^*
Blood pressure				NS
Systolic (mmHg)	121.7 (12.1)	120.6 (12.4)	122.2 (9.4)	
Diastolic (mmHg)	79.8 (30.1)	77.8 (9.0)	79.3 (7.7)	NS
Pregestational weight (kg)	76.4 (17.8)	78.4 (20.2)	80.8 (25.9)	NS
Pregestational BMI (kg/m^2^)	27.8 (6.5)	29.3 (6.7)	30.8 (8.8)	.034*^b^*
Underweight (BMI <18.5), n (%)	13 (2.1)	2 (1.5)	0.0 (0.0)	NS
Normal weight (BMI ≥18.5-<25), n (%)	159 (26.2)	27 (19.7)	5 (25.0)	
Overweight (BMI ≥25-<30), n (%)	142 (23.4)	33 (24.1)	4 (20.0)	
Obese (BMI ≥30), n (%)	145 (23.8)	41 (29.9)	9 (45.0)	
Missing, n (%)	149 (24.5)	34 (24.8)	2 (10.0)	
Gestational weight gain (kg)	10.0 (6.4)	10.5 (5.2)	8.4 (7.3)	NS
Underweight (pregestational BMI <18.5) (kg)	14.4 (6.7)	23.5 (0.0)	0.0 (0.0)	NS
Normal weight (pregestational BMI ≥18.5-<25) (kg)	12.6 (5.3)	11.1 (3.8)	8.4 (4.3)	NS
Overweight (pregestational BMI ≥25-<30) (kg)	10.4 (6.6)	10.6 (5.3)	9.9 (7.5)	NS
Obese (pregestational BMI ≥30) (kg)	6.9 (5.7)	10.0 (5.6)	7.6 (9.3)	.038*^b^*
Followed the recommendations regarding GWG set by IOM				.028*^c^*
Yes, n (%)	139 (22.9)	33 (24.1)	4 (20.0)	
No, gained more weight, n (%)	131 (21.5)	40 (29.2)	3 (15.0)	
No, gained less weight, n (%)	159 (26.2)	25 (18.2)	10 (50.0)	
Missing, n (%)	179 (29.4)	39 (28.5)	3 (15.0)	
Insulin treated during pregnancy, n (%)	46 (7.6)	30 (21.9)	7 (35.0)	.02*^c^*, <.001*^d^*
Insulin dose (daily dose, IU)	25.5 (12.4)	35.7 (25.1)	60.6 (23.9)	<.001*^b^*, .004*^d^*
Insulin dose (IU/kg)	0.28 (0.13)	0.37 (0.21)	0.75 (0.32)	.003*^c^*, <.001*^d^*
**Biochemical parameters**				
C-peptide (370-1470 pmol/L)	2235.2 (1296.2)	2070.4 (1300.4)	2035.0 (1123.9)	NS
Anti-Gad65ab (10^3^ int. U/L):				NS
Anti-Gad65ab (10^3^ int. U/L) <10, n (%)	542 (89.1)	123 (89.8)	19 (95.0)	
Anti-Gad65ab (10^3^ int. U/L) >10, n (%)	14 (2.3)	4 (2.9)	0 (.0)	
Missing, n (%)	52 (8.6)	10 (7.3)	1 (5.0)	
HOMA-B	358.5 (228.9)	314.9 (156.5)	292.3 (156.4)	NS
HOMA-IR	6.2 (3.9)	5.9 (3.8)	5.7 (3.2)	NS
Plasma glucose (4.2-7.8 mmol/L)	5.3 (1.2)	5.5 (1.0)	5.6 (1.4)	NS
HbA1c (<48 mmol/mol)	34.7 (4.0)	37.2 (5.2)	37.9 (6.1)	.004*^d^*, < .001*^b^*
B-hemoglobin (7.0-9.1 mmol/L)*^a^*	7.7 (0.9)	7.6 (0.9)	7.7 (0.9)	NS
Triglyceride (1.33-4.72 mmol/mol)*^a^*	3.4 (1.2)	3.4 (1.2)	3.8 (1.5)	NS
Cholesterol				
Total cholesterol (4.4-8.8 mmol/L)*^a^*	6.2 (1.1)	5.9 (1.2)	5.7 (1.1)	.004*^b^*
HDL (1.2-2.9 mmol/L)*^a^*	1.7 (0.4)	1.7 (0.4)	1.7 (0.3)	NS
LDL (1.6-5.6 mmol/L)*^a^*	3.0 (1.0)	2.9 (1.1)	2.7 (.8)	NS
CRP (< 8.0 mg/L)	12.5 (20.0)	13.9 (28.1)	26.1 (31.9)	NS
ALAT (5-42 U/I)*^a^*	30.1 (42.3)	28.9 (37.2)	16.8 (10.4)	NS
**Background data**				
Education				.009*^d^*
Elementary school, n (%)	25 (4.1)	9 (6.6)	0 (.0)	
Short-term education, n (%)	162 (26.6)	46 (33.6)	6 (30.0)	
Intermediate higher education, n (%)	220 (36.2)	46 (33.6)	6 (30.0)	
Long higher education, n (%)	164 (27.0)	27 (19.7)	2 (10.0)	
Other (including foreign education), n (%)	17 (2.8)	6 (4.4)	4 (20.0)	
Missing, n (%)	20 (3.3)	3 (2.2)	2 (10.0)	
Ethnicity				.004*^d^*, <.001*^c^*
Europe, n (%)	493 (81.1)	86 (62.8)	10 (50.0)	
Asia, n (%)	29 (4.8)	18 (13.1)	1 (5.0)	
Middle east, n (%)	55 (9.0)	19 (13.9)	5 (25.0)	
Other, n (%)	10 (1.6)	11 (8.0)	2 (10.0)	
Missing, n (%)	21 (3.5)	3 (2.2)	2 (10.0)	
Parity				NS
Primipara, n (%)	296 (48.7)	65 (47.4)	6 (30.0)	
Multipara, n (%)	294 (48.4)	69 (50.4)	12 (60.0)	
Missing, n (%)	18 (3.0)	3 (2.2)	2 (10.0)	
Family history of diabetes mellitus*^e^*				<.001*^b^*
Yes, n (%)	228 (37.5)	69 (50.4)	4 (20.0)	
No, n (%)	266 (43.8)	36 (26.3)	11 (55.0)	
Missing, n (%)	114 (18.8)	32 (23.4)	5 (25.0)	
Physical activity*^f^*				NS
Never physical active, n (%)	43 (7.1)	12 (8.8)	1 (5.0)	
Physical active 1-3 days/week, n (%)	150 (24.7)	29 (21.2)	0 (.0)	
Physical active 4-7 days/week, n (%)	156 (25.7)	43 (31.4)	4 (20.0)	
Missing, n (%)	259 (42.6)	53 (38.7)	15 (75.0)	
Smoking				NS
Never smoked, n (%)	275 (45.2)	56 (40.9)	5 (25.0)	
Smoked before pregnancy, n (%)	54 (8.9)	16 (11.7)	0 (0.0)	
Smoking, n (%)	18 (3.0)	10 (7.3)	0 (0.0)	
Missing, n (%)	261 (42.9)	55 (40.1)	15 (75.0)	

Data are mean (SD) unless otherwise indicated. *P* values calculated within the 3 glycemic groups: normoglycemic, pDM, and T2DM. A total of 765 women are described in this table. This corresponds to the total number of women participating at follow-up, excluding the 3 women diagnosed with T1DM.

Abbreviations: ALAT, alanine transaminase; B, β-cell function; BMI, body mass index; CRP, C-reactive protein; HDL, high-density lipoprotein; HOMA, homeostatic model assessment; IR, insulin resistance; pDM; prediabetes mellitus; T1DM, type 1 diabetes; T2DM, type 2 diabetes.

^*a*^Reference interval for pregnant women GW 35-42 [16]. One-way analysis of variance was used to compare groups and the Student t-test for post hoc analysis.

^*b*^Statistical significant difference between normoglycemic and pDM.

^*c*^Statistical significant difference between pDM and T2DM.

^*d*^Statistical significant difference between normoglycemic and T2DM.

^*e*^Family history of diabetes defined as 1 or more relatives with DM (for further details, see text).

^*f*^Physical activity defined as at least 30 minutes pr day (for further details, see text).

When comparing the clinical baseline characteristics, based on the glycemic status of the women at follow-up, there was a significant difference in the pregestational BMI between the NG group and the pDM group: 27.8 kg/m^2^ (6.5) vs 29.3 kg/m^2^ (6.7); *P* = .034; and between the NG group and the T2DM group: 27.8 kg/m^2^ (6.5) vs 30.8 kg/m^2^ (8.8) (Table 2). When looking at GWG in the group of women with a pregestational BMI > 30 kg/m^2^, those who were pDM at follow-up had gained significantly more weight during pregnancy than the women in the NG group (6.9 kg [5.7] vs 10.0 kg [5.6], respectively; *P* < .038) (Table 2).

Considering GWG by IOM recommendations, we found a significant difference in the distribution among the women with pDM and T2DM, as 50% of the women later diagnosed with T2DM had gained less weight than recommended compared with 18% among women later categorized with pDM (*P* = .028). The distribution among the NG and pDM groups was equal with 20% to 25% that either reached the recommended weight gain by IOM, gained more, or gained less weight (*P* = .058) (Table 2).

Regarding insulin treatment late in pregnancy, 35% of the women who were later diagnosed with T2DM were treated with insulin compared with 22% of the women who were later diagnosed with pDM (*P* = .02) and compared with only 8% of the women who stayed NG within the first year PP (*P* = <.001). Concerning daily insulin dose (IU), there was also a significant difference between the NG and the pDM groups (25.5 [12.4] vs 35.7 [25.1]; *P* ≤ .001) and between the pDM and the T2DM groups (35.7 [25.1] vs 60.6 [23.9]; *P* = .004). Thus, the women who needed the highest insulin doses were the ones who later developed T2DM. The average amount of insulin per kilogram (IU/kg) was also significantly higher in the T2DM group than in the pDM group (0.37 [0.21] vs 0.75 [0.32]; *P* = .003) and compared with the NG group (0.28 [0.13] vs 0.75 [0.32]; *P* ≤ .001) (Table 2).

### Baseline Biochemical Parameters Divided According to Glycemic Status at Follow-up (OGTT 3 Months PP or 1 Year PP)

In the group of women who were diagnosed with pDM and T2DM during follow-up, HbA1c values were already higher at baseline than in the women who were later categorized as NG (37.2 mmol/mol [5.2] vs 34.7 mmol/mol [4.0]; *P* ≤ .001 and 37.9 mmol/mol [6.1] vs 34.7 mmol/mol [4.0]; *P* = .004). In contrast to this finding, women with pDM at follow-up presented with lower levels of total cholesterol at baseline than the women who were NG at follow-up (5.9 mmol/L [1.2] vs 6.2 mmol/L [1.1]; *P* = .004). Levels of C-peptide, HOMA-B, and HOMA-IR as well as CRP, hemoglobin, plasma glucose, triglycerides, HDL and LDL cholesterol, and ALAT were not found to be different among the 3 groups at the GW 34-38 visit. In addition, there was no significant difference in diastolic or systolic BP among groups.

### Baseline Socioeconomic Data Divided According to Glycemic Status at Follow-up (OGTT 3 Months PP or 1 Year PP)

A notable disparity in educational attainment was observed between women in the NG group and those with T2DM. Among the women in the NG group, a substantial majority of 63% possessed an educational background consisting of either intermediate or long duration studies, whereas only 40% of the women later diagnosed with T2DM had an intermediate- and long education (*P* = .009). Most of the NG women were of European descent (81%) contrasted with 63% in the pDM group (*P* ≤ .001) and 50% in the T2DM group (*P* = .004). Finally, a larger proportion of women in the pDM group had a family history of diabetes (50%) compared with NG women (38%) (*P* ≤ .001). We found no difference in parity, level of physical activity, or smoking among the groups.

### Clinical Characteristics Either at Follow-up OGTT 3 Months PP or at 1 Year PP Visit Depending on Glycemic Status.

Table 3 represent clinical characteristics either at follow-up OGTT 3 months PP or at 1 year PP divided into glycemic groups (NG, pDM, and T2DM). Women with pDM had the highest BMI when compared to women in the NG group (30.1 kg/m^2^ vs 27.6 kg/m^2^; *P* ≤ .001) and both C-peptide levels, fasting glucose, HbA1c, triglycerides, total cholesterol, and HOMA-IR increased with increasing levels of glycemia.

**Table 3.** Clinical characteristics at either follow-up OGTT 3 months postpartum or at the 1 year PP visit depending on glycemic status (n = 765)

	Normoglycemic (n = 608)	pDM (n = 137)	T2DM (n = 20)	Between groups
Weight at 1-year follow-up (kg)	75.9 (18.2) (n = 310)	80.2 (21.3) (n = 67)	74.0 (16.9) (n = 9)	NS
Weight change 1 year postpartum				NS
Reached pregestational weight, n (%)	62 (10.2)	8 (5.8)	2 (10.0)	
Lost weight compared with pregestational weight, n (%)	123 (20.2)	19 (13.9)	5 (25.0)	
Gained weight compared with pregestational weight, n (%)	125 (20.6)	40 (29.2)	2 (10.0)	
Missing, n (%)	298 (49.0)	70 (51.1)	11 (55.0)	
BMI (kg/m^2^)	27.6 (6.0)	30.1 (7.1)	28.8 (6.6)	<.001*^a^*
Hip/waist ratio	1.2 (0.1)	1.2 (0.1)	1.2 (0.1)	NS
Blood pressure:				
Systolic (mmHg)	117.1 (13.8)	117.5 (11.5)	124.8 (22.4)	NS
Diastolic (mmHg)	79.0 (10.3)	77.1 (8.1)	81.5 (12.6)	NS
**Biochemical parameters**				
C-peptide (370-1470 pmol/L)	851.9 (496.7)	1233.9 (978.1)	1357.1 (714.4)	<.001*^a^*, <.001*^c^*
Anti-Gad65ab (10^3^ int. U/L):				NS
Anti-Gad65ab (10^3^ int. U/L) <10, n(%)	337 (55.4)	119 (86.9)	18 (90.0)	
Anti-Gad65ab (10^3^ int. U/L) >10, n(%)	10 (1.6)	4 (2.9)	0 (.0)	
Missing, n(%)	261 (42.9)	14 (10.2)	2 (10.0)	
HOMA-B	131.4 (76.4)	135.8 (81.2)	116.5 (102.0)	NS
HOMA-IR	2.9 (0.7)	4.1 (1.4)	5.7 (2.7)	<.001*^a^*, <.001*^c^*
Plasma glucose (4.2-7.8 mmol/L)	5.2 (0.4)	6.0 (1.1)	6.3 (1.8)	<.001*^a^*, <.001*^c^*
HbA1c (<48 mmol/mol)	34.5 (3.0)	36.9 (4.4)	40.1 (8.2)	<.001*^c^*, .028^b^
B-hemoglobin (7.3-9.5 mmol/L)	8.3 (0.6)	8.3 (0.5)	8.3 (0.6)	NS
Triglyceride (< 3.0 mmol/mol)	1.1 (0.6)	1.3 (0.7)	2.1 (1.6)	<.001^c^, .026^b^
Cholesterol				
Total cholesterol (<5.0 mmol/L)	4.6 (.8)	4.7 (1.0)	4.9 (0.9)	.041^a^
HDL (>1.2 mmol/L)	1.4 (0.3)	1.3 (0.3)	1.3 (0.4)	NS
LDL (<3.0 mmol/L)	2.8 (0.7)	2.8 (0.8)	2.8 (0.4)	NS
CRP (<8.0 mg/L)	8.1 (8.1)	10.1 (5.2)	4.3 (2.3)	NS
ALAT (10-45 U/I)	21.1 (13.3)	27.1 (40.4)	12.6 (2.6)	NS

Data are mean (SD) unless otherwise indicated. The data displayed are the values recorded at time of diagnosis of dysglycemia, n = indicates number of women with accessible data. If the woman attended both OGTT 3 months postpartum and 1-year follow-up and were normoglycemic at both visits, data are recorded from the 1-year follow-up. One-way ANOVA was used to compare groups and Student t-test for post hoc analysis. *P* values calculated within the 3 glycemic groups: normoglycemic, pDM and T2DM

A total of 765 women are described in this table. This corresponds to the total number of women participating at follow-up, excluding the 3 women diagnosed with T1DM.

Abbreviations: ALAT, alanine transaminase; B, β-cell function; BMI, body mass index; CRP, C-reactive protein; HDL, high-density lipoprotein; HOMA, homeostatic model assessment; IR, insulin resistance; LDL, low-density lipoprotein; OGTT, oral glucose tolerance text; pDM; prediabetes mellitus; PP, postpartum; T1DM, type 1 diabetes; T2DM, type 2 diabetes.

^*a*^Statistically significant difference between normoglycemic and pDM.

^*b*^Statistical significant difference between pDM and T2DM.

^*c*^Statistical significant difference between normoglycemic and T2DM.

Comparing the 3 groups regarding measurements performed at the OGTT or the 1-year follow-up, fasting C-peptide levels were higher among women in the pDM- and T2DM group, compared to the NG women: 1233.9 pmol/L (978.1) vs 851.9 pmol/L (496.7); *P* ≤ .001 and 1357.1 pmol/L (714.4) vs 851.9 pmol/L (496.7); *P* ≤ .001. A similar tendency was seen in HOMA-IR as insulin resistance was significantly lower in the NG group compared to the pDM- and T2DM groups (2.9 [0.7] vs 4.1 (1.4); *P* ≤ .001 and 2.9 [0.7] vs 5.7 [2.7]; *P* ≤ .001). Higher values of triglycerides were seen in women with T2DM compared with NG and pDM, when comparing measurements from either OGTT or 1-year follow-up (2.1 mmol/mol [1.6] vs 1.1 mmol/mol [0.6]; *P* ≤ .001 and 2.1 mmol/mol [1.6] vs 1.3 mmol/mol [0.7], *P* = .026). This was also the case with HbA1c (40.1 mmol/mol [8.2] vs 34.5 mmol/mol [3.0]; *P* ≤ .001 and 40.1 mmol/mol [8.2] mmol/mol vs 36.9 mmol/mol [4.4]; *P* = .028).

As weight influences the likelihood of developing T2DM following GDM, analyses were performed to determine whether participants returned to their pregestational weight 1 year PP. As seen in Table 3, 10.2% of NG, 5.8% of pDM, and 10.0% of T2DM women reached their pregestational weight. Among the women who lost weight, 20.2% were NG, 13.9% had pDM, and 25.0% had T2DM 1 year PP. Lastly, of the women who gained weight, 20.6% were NG, 29.2% were pDM, and 10.0% were T2DM. In total, 18.7% of women returned to their pregestational weight, 38.1% lost weight, and 43.3% had gained weight 1 year PP.

As the follow-up program was voluntary, there was a large number of women who did not attend the visits PP. Therefore, baseline data from the women who did not participate in the follow-up program were compared with the baseline data from the women who did participate (Table 4). The nonparticipating women were found to be younger, had a higher pregestational weight but similar BMI, had lower HDL cholesterol levels, had a shorter educational background, and were more often of non-European descent.

**Table 4.** Drop-out analysis

	Participated in either 3 months or 1 year PP visit n = 768	Did not participate in either 3 months or 1 PP visit n = 505	*P* value
Age (years)	34.0 (4.9)	33.2 (4.9)	.003*^b^*
Gestation week at pregnancy visit (week)	36.3 (1.9)	36.3 (2.0)	.857
Blood pressure			
Systolic (mmHg)	121.5 (0.5)	121.5 (0.6)	.977
Diastolic (mmHg)	79.4 (1.0)	79.0 (.4)	.760
Pregestational weight (kg)	76.9 (18.5)	79.3 (19.7)	.042*^b^*
Pregestational BMI (kg/m^2^)	28.0 (6.3)	28.5 (6.6)	.353
Pregestational BMI (kg/m^2^)			.689
Underweight (BMI <18.5), n (%)	15 (2.0)	5 (1.0)	
Normal weight (BMI ≥18.5-<25), n (%)	191 (25.0)	91 (18.0)	
Overweight (BMI ≥25-<30), n (%)	179 (23.4)	98 (19.4)	
Obese (BMI ≥ 30), n (%)	195 (25.5)	92 (18.2)	
Missing, n (%)	185 (24.1)	219 (43.4)	
Gestational weight gain (kg)	10.1 (6.2)	10.0 (9.6)	.942
Underweight (pregestational BMI <18.5) (kg)	15.2 (2.0)	13.8 (3.7)	.733
Normal weight (BMI ≥18.5-<25) (kg)	12.3 (.4)	13.3 (.7)	.187
Overweight (BMI ≥25-<30) (kg)	10.5 (.5)	10.6 (.8)	.831
Obese (BMI ≥ 30) (kg)	7.6 (.4)	6.3 (1.6)	.279
Followed the recommendations regarding GWG set by IOM:			.469
Yes, n (%)	176 (22.9)	90 (17.8)	
No, gained more weight, n (%)	174 (22.7)	95 (18.8)	
No, gained less weight, n (%)	194 (25.2)	86 (17.1)	
Missing, n (%)	224 (29.9)	234 (46.3)	
**Biochemical parameters**			
C-peptide (370-1470 pmol/L)	2192.7 (64.0)	2090.1 (70.6)	.327
Anti-Gad65ab (10^3^ int. U/I)			.193
Anti-Gad65ab (10^3^ int. U/I) <10, n (%)	684 (89.1)	391 (77.4)	
Anti-Gad65ab (10^3^ int. U/I) >10, n (%)	21 (2.7)	14 (2.8)	
Missing, n (%)	63 (8.2)	100 (19.8)	
HOMA-B	347.2 (11.0)	351.1 (17.7)	.846
HOMA-IR	6.1 (0.2)	5.6 (0.2)	.099
Plasma-glucose (4.2-7.8 mmol/L)	5.5 (0.1)	5.5 (0.1)	.641
HbA1c (<48 mmol/mol)	35.3 (0.2)	35.7 (0.2)	.089
Hemoglobin (7.0-9.1 mmol/L)*^a^*	7.7 (0.0)	7.7 (0.0)	.524
Triglyceride (1.33-4.72 mmol/mol)*^a^*	3.4 (0.1)	3.3 (0.1)	.412
Cholesterol			
Total cholesterol (4.4-8.8 mmol/L)*^a^*	6.2 (0.1)	6.0 (0.1)	.074
HDL (1.2-2.9 mmol/L)*^a^*	1.7 (0.0)	1.6 (0.0)	<.001*^b^*
LDL (1.6-5.6 mmol/L)*^a^*	3.0 (0.0)	3.0 (0.1)	.859
CRP (<8 mg/L)	13.2 (1.1)	15.9 (1.9)	.181
ALAT (5-42 U/I)*^a^*	29.5 (1.6)	26.3 (1.6)	.176
**Background data**			
Education			<.001*^b^*
Elementary school, n (%)	34 (4.4)	28 (5.5)	
Short-term education, n (%)	214 (28.0)	165 (32.7)	
Intermediate higher education, n (%)	272 (35.6)	153 (30.3)	
Long higher education, n (%)	193 (25.2)	75 (14.9)	
Other (including foreign education), n (%)	27 (3.5)	15 (3.0)	
Missing, n (%)	25 (3.3)	69 (13.7)	
Ethnicity			.021*^a^*
Europe, n (%)	589 (77.0)	371 (73.5)	
Asia, n (%)	48 (6.3)	27 (5.3)	
Middle east, n (%)	79 (10.3)	24 (4.8)	
Other, n (%)	23 (3.0)	11 (2.2)	
Missing, n (%)	26 (3.4)	72 (14.3)	
Parity			.858
Primipara, n (%)	367 (48.0)	219 (43.4)	
Multipara, n (%)	375 (49.0)	219 (43.4)	
Missing, n (%)	23 (3.0)	67 (13.3)	
Family history of diabetes mellitus*^c^*			.165
Yes, n (%)	308 (40.3)	158 (31.3)	
No, n (%)	306 (40.0)	192 (38.0)	
Missing, n (%)	151 (19.7)	155 (30.7)	
Physical activity*^d^*			.272
Never physical active, n (%)	56 (7.3)	9 (1.8)	
Physical active 1-3 days/week, n (%)	179 (23.4)	14 (2.8)	
Physical active 4-7 days/week, n (%)	203 (26.5)	22 (4.4)	
Missing, n (%)	327 (42.7)	460 (91.1)	
Smoking			<.001*^a^*
Never smoked, n (%)	336 (43.9)	26 (5.1)	
Smoked before pregnancy, n (%)	70 (9.2)	8 (1.6)	
Smoking, n (%)	28 (3.7)	10 (2.0)	
Missing, n (%)	331 (43.3)	461 (91.3)	

Data are mean (SD) unless otherwise indicated.

Abbreviations: ALAT, alanine transaminase; B, β-cell function; BMI, body mass index; CRP, C-reactive protein; HDL, high-density lipoprotein; HOMA, homeostatic model assessment; IR, insulin resistance; LDL, low-density lipoprotein; OGTT, oral glucose tolerance text; PP, postpartum.

^*a*^Reference interval for pregnant women GW 35-42 [16]. For numerical variables *P*-values was generated using Student t-test. For categorical variables, a chi^2^ test was performed.

^*b*^ *P* values that are statistically significantly different.

^*c*^Family history of diabetes defined as 1 or more relatives with DM (for further details, see text).

^*d*^Physical activity defined as at least 30 minutes pr day (for further details, see text).

## Discussion

In an attempt to evaluate glycemic and metabolic status within the first year PP in pregnancies complicated by GDM, we found, that in a clinical setting, almost 21% of women with GDM who volunteered to be part of a follow-up program had developed either pDM, T2DM, or T1DM within the first year PP. Metabolically, we found significantly higher values of C-peptide, HOMA-IR, FPG, HbA1c, triglyceride, and total cholesterol among women with pDM and T2DM than NG women up to 1 year PP. Regarding weight change, more than 40% of the women gained weight in the first year PP, whereas almost 40% managed to lose weight compared with their pregestational weight. Comparing baseline characteristics, we found significant differences in pregestational BMI, as the women who later developed T2DM had a higher pregestational BMI than the women with pDM and NG. In addition, the women who developed pDM or T2DM had a higher HbA1c late in pregnancy than the women who stayed NG within the first year PP. Regarding GWG, the majority of women later diagnosed with T2DM gained weight less than recommended by the IOM. This could be because these women had a higher weight and a higher BMI prior to pregnancy, possibly making the women more careful not to gain weight and making the dieticians more attentive to give advice on the IOM recommendations. At follow-up 1 year PP, women with pDM had a significantly higher BMI than women with NG and women with T2DM. The surprising finding that women with NG and T2DM did not differ in BMI could be explained by the small number of women with T2DM with weight data available (n = 9 with T2DM and n = 310 with NG).

A primary aim of our study was to make a metabolic characterization of women with GDM in terms of glycemic parameters and lipid profiles both late in pregnancy and during the first year PP in order to identify the women who might have the highest risk of developing diabetes after GDM. Other studies have previously performed subclassifications of women with GDM, such as Powe et al [7], who defined physiological subtypes of GDM using the Matsuda index [17-19] based on an OGTT in pregnancy. Of 809 women, 67 women developed GDM, and these women were divided into groups consisting of GDM with a predominant insulin sensitivity defect (GDM sensitivity), a predominant insulin secretion defect (GDM secretion) or a GDM with both defects (mixed GDM). Although we were not able to perform the Matsuda index as Powe et al [7], we sought to estimate insulin resistance using HOMA-IR, based on blood glucose and C-peptide and HOMA-B to assess insulin secretion. HOMA-IR and HOMA-B were evaluated late in pregnancy (GW 34-38) in all the women with GDM and were compared according to their glycemic status within the first year PP (NG, pDM, T2DM) both late in pregnancy and again at time of diagnosis of pDM or T2DM. C-peptide levels late in pregnancy were significantly above normal range in all 3 groups, compatible with the pronounced insulin resistance seen in late pregnancy. However, due to ethical reasons, the women in our study were not fasting when blood samples were collected in pregnancy, which can also explain the high levels of C-peptide in all 3 groups. HOMA-IR and HOMA-B were similar between the groups in pregnancy and could thus not predict who would develop pDM or T2DM within the first year PP. Again, the HOMA-IR and HOMA-B calculations based on C-peptide and blood glucose measured in pregnancy should be interpreted with caution as the HOMA equations are based on fasting values, although correlations between fasting and non-fasting blood tests to assess insulin resistance have been proven to be strong [20]. As expected, fasting C-peptide levels and HOMA-IR were higher among women in the pDM and T2DM groups than in the women with NG when the diagnosis pDM or T2DM was given PP.

Just as HOMA-IR is a surrogate marker of insulin resistance, insulin requirements can to some extent express the degree of insulin resistance. Thus, the women who developed pDM and T2DM within the first year PP needed significantly more insulin than the ones who stayed normoglycemic. Accordingly, insulin needs late in pregnancy could be an indicator of who will later develop diabetes.

In addition to the higher insulin requirements, HbA1c levels were significantly higher late in pregnancy in the women who developed T2DM within the first year PP. This has also been found in a study by Jotic et al [21], who aimed to determine the prevalence and predictors for prediabetes among women with previous GDM. Out of 186 women with previous GDM, 43% developed pDM within the first 4-12 weeks PP. In the third trimester of pregnancy, HbA1c was higher in women with pDM 4-12 weeks PP, but fasting blood glucose were comparable. This was similar to the findings in our study. HbA1c late in pregnancy could thus potentially be used to predict who will develop diabetes after GDM.

In the study by Jotic et al [21], lipid profiles were also evaluated in the third trimester of pregnancy and the authors found that the women who developed pDM had higher levels of total cholesterol, triglycerides, and LDL, whereas HDL levels were lower. In our study, there was no difference in lipids late in pregnancy when comparing the 3 groups, apart from total cholesterol levels, which surprisingly were lower in the group of women who later developed T2DM. However, PP triglycerides and total cholesterol levels were significantly higher in the women with T2DM at time of diagnosis than in the women with NG and pDM. The same tendency was observed in a cohort of 67 women with GDM [9], in which the most insulin-resistant women had the highest levels of triglycerides and lowest levels of HDL [9]. Again, it must be kept in mind that the women in the current study were not fasting when the blood samples were taken during pregnancy, but as shown by Mora et al [22] nonfasting lipid levels are similar to fasting lipid levels. The reference values for lipids late in pregnancy are, however, higher than nonpregnancy values. Thus, the higher lipid values among the pregnant women in our study is due to pregnancy and not due to the nonfasting state [16].

A strength of our study is the large number of women with GDM included. Furthermore, our study reflect a daily clinical setting, describing the heterogeneity of women with GDM.

There are however some limitations to our study. A major limitation is the huge drop out number. Two explanations for this may prevail: First, the prevention program was voluntary and even though the participants received reminders about their appointments, some did not show up and others cancelled their appointments for various reasons. Second, the Covid-19 pandemic ranging from 2020 to 2022 demanded isolation and visits at the hospitals were limited, especially for pregnant women, resulting in a large dropout rate as healthy pregnant women were advised to avoid hospitals. The drop-out analysis showed that the women who did not attend the PP follow-up were younger, had a higher pregestational weight (but similar BMI), were more often of non-European descent, and had a lower education level than the women who attended follow-up 1 year PP. The 2 groups did however not differ regarding third trimester biochemical parameters. Seeing a difference in both age, ethnicity, education level, and pregestational weight between the group of women who did and did not attend the PP follow-up results in a selection bias, but also draws attention to the problem that there is a group of younger and lower educated women, some from ethnic minorities, who are difficult to reach with the current prevention programs. In a study by Benhalima et al [23], including 191 women with GDM, only 29.3% did not attend the scheduled OGTT 3 months PP. The women who did not attend the OGTT were somewhat similar in our “drop-out cohort” to the women in Benhalima's study, who did not attend the OGTT, had a higher BMI, were more often from an ethnic minority, and were more likely to be smokers. Interestingly there was a very high number of women with dysglycemia in Benhalima's study 3 months PP as 42.2% had pDM and 1.5% had overt diabetes. In comparison, we found that 1 year PP only 20.8% had pDM or overt diabetes. This could indicate that our clinical follow-up program, including individual counseling on how to prevent future diabetes could have had an effect. It is however impossible to make a solid conclusion as our study was not a randomized trial. Important to keep in mind is also that the current project was a voluntary follow-up program and as the group who did not participate was younger, had a higher BMI, and a lower education it could be speculated that they would have an even higher risk of dysglycemia, leading to an underestimation of the true prevalence of pDM and overt diabetes.

Another disadvantage of the study is the short follow-up time. The study population was reviewed within the first year after childbirth, whereas other studies report PP assessment after 5 to 25 years [3, 24, 25]. It could be argued that the follow-up period of 1 year is not sufficiently long enough to review the glycemic status of women with prior GDM. However, it is still of value to know how many women with prior GDM will develop diabetes or prediabetes within the first year in a large, primarily European, cohort.

Despite several studies demonstrating strong evidence that achieving pregestational weight and PP weight loss can help prevent diabetes diagnosis after GDM [24-26], there was no significant difference observed in the distribution of glycemic status based on weight status (see Table 3). This may be due to the short follow-up period or because there was a lot of missing data on weight, reducing the number of women and power, regarding that outcome.

Most participants were of European origin, limiting generalizability, and although the cohort was quite large, the sample size of women with T2DM 1 year after GDM was small, also contributing to limited power.

Despite the limitations, we conclude from this large clinical study that out of 1270 women with GDM, 768 (60%) voluntarily attended at least 1 follow-up visit within the first year PP. Of these 768 women, 20.8% had either pDM, T2DM, or T1DM within the first year PP. Almost half of the women gained weight within the first year PP.

In a daily clinical practice women with GDM are considered to have the same disease and are treated according to the same protocols. We demonstrate a heterogeneity among women with GDM, why health systems are encouraged to personalize the risk assessment and individualize therapy and prevention strategies regarding weight loss programs and targeting women of shorter education and with different ethnic backgrounds to prevent T2DM after GDM. Thus, it is highly relevant to elucidate the heterogeneity of GDM as this will enable clinicians to tailor the best possible surveillance program for the individual woman with previous GDM.

## Data Availability

The datasets used and/or analyzed during the current study are available from the corresponding author on reasonable request.

## Abbreviations

ALAT: alanine transaminase
ANOVA: analysis of variance
B: β-cell function
BMI: body mass index
BP: blood pressure
CRP: C-reactive protein
FPG: fasting plasma glucose
GDM: gestational diabetes mellitus
GW: gestational week
GWG: gestational weight gain
HDL: high-density lipoprotein
HOMA: homeostatic model assessment
IR: insulin resistance
IU: international units
LDL: low-density lipoprotein
NG: normoglycemia
OGTT: oral glucose tolerance test
PP: postpartum
T1DM: type 1 diabetes
T2DM: type 2 diabetes
